# Long-term potentiation reconstituted with an artificial TARP/PSD-95 complex

**DOI:** 10.1016/j.celrep.2022.111483

**Published:** 2022-10-11

**Authors:** Anagh Sinha Ravi, Menglong Zeng, Xudong Chen, Gerardo Sandoval, Javier Diaz-Alonso, Mingjie Zhang, Roger A. Nicoll

**Affiliations:** 1 Department of Cellular and Molecular Pharmacology, University of California at San Francisco, San Francisco, CA, USA; 2 Division of Life Science, Hong Kong University of Science and Technology, Clear Water Bay, Kowloon, Hong Kong, China; 3 Department of Anatomy and Neurobiology, University of California at Irvine, Irvine, CA, USA; 4 Center for the Neurobiology of Learning and Memory, University of California at Irvine, Irvine, CA, USA; 5 Greater Bay Biomedical Innocenter, Shenzhen Bay Laboratory, Shenzhen 518036, China; 6 School of Life Sciences, Southern University of Science and Technology, Shenzhen 518055, China; 7 Lead contact

## Abstract

The critical role of AMPA receptor (AMPAR) trafficking in long-term potentiation (LTP) of excitatory synaptic transmission is now well established, but the underlying molecular mechanism is still uncertain. Recent research suggests that PSD-95 captures AMPARs via an interaction with the AMPAR auxiliary subunits—transmembrane AMPAR regulatory proteins (TARPs). To determine if such interaction is a core minimal component of the AMPAR trafficking and LTP mechanism, we engineered artificial binding partners, which individually were biochemically and functionally dead but which, when expressed together, rescue binding and both basal synaptic transmission and LTP. These findings establish the TARP/PSD-95 complex as an essential interaction underlying AMPAR trafficking and LTP.

## In brief

Synaptic scaffolding protein PSD-95 captures AMPARs via an interaction with the AMPAR auxiliary subunits—TARPs. To determine the centrality of the TARP/PSD-95 interaction, Ravi et al. engineered binding partners that are biochemically and functionally dead individually. However, when expressed together, they rescue TARP/PSD-95 binding, basal AMPAR mediated synaptic transmission, and LTP.

## Graphical Abstract

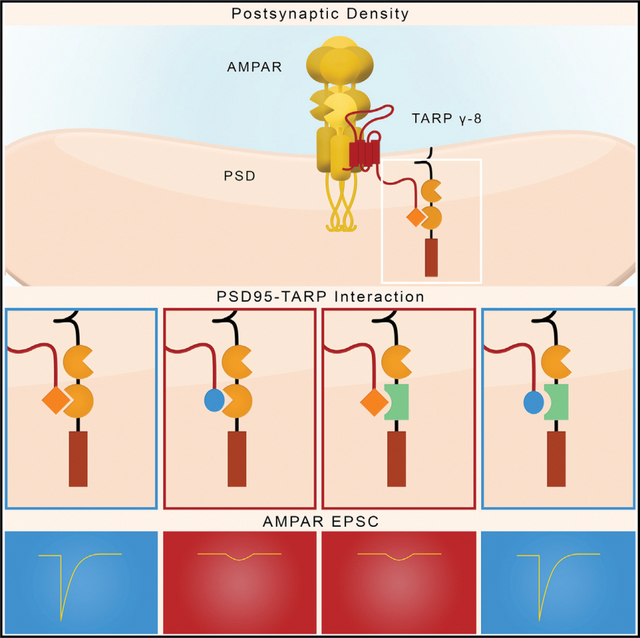

## INTRODUCTION

It is now well accepted that long-term potentiation (LTP), in which brief repetitive stimulation results in a long-lasting increase in synaptic strength, is mediated primarily by the activity dependent synaptic accumulation of AMPA receptors (AMPARs). Much of the current research on LTP focuses on the mechanisms by which NMDA receptor (NMDAR) activation results in this AMPAR accumulation. AMPARs contain cytoplasmic carboxy-terminal domains (CTDs) that have been proposed to be an essential target of the LTP signal (Hayashi et al., 2000; Huganir and Nicoll, 2013; Malinow and Malenka, 2002; Nicoll, 2017; Shi et al., 2001). However, the ability to evoke normal LTP in the absence of AMPAR CTDs (Diaz-Alonso et al., 2020; Granger et al., 2013; Watson et al., 2021) casts doubt on this proposal. In addition, a family of auxiliary proteins termed transmembrane AMPAR regulatory proteins (TARPs) assemble with AMPARs (Greger et al., 2017; Jackson and Nicoll, 2011; Schwenk et al., 2012; Straub and Tomita, 2012), and these have also been proposed to be a target of the LTP signal. TARPs contain a CTD, which is proposed to serve two functions. First, a proximal domain is a target of covalent modification and is proposed to be important for the delivery of AMPARs during LTP (Hafner et al., 2015; Opazo et al., 2012; Park et al., 2016; Sumioka et al., 2010; Tomita et al., 2005). Recent studies have reexamined the role of the TARP CTD proximal domain in basal synaptic transmission and LTP (Sheng et al., 2018; Zeng et al., 2019) and found that normal LTP can be evoked in the absence of the covalent modification. Although the basis for the differences between these studies is unclear, it is possible that differences in experimental strategies, such as receptor subunit composition, could play a role.

Second, the TARP CTD contains a terminal PDZ binding motif (PBM) that binds to the PDZ domains of synaptic scaffolding proteins, such as PSD-95. Removal of the TARP PBM eliminates synaptic targeting and LTP (Sheng et al., 2018; Zeng et al., 2019).

How are AMPAR/TARP complexes held at the synapse? Most attention has focused on a family of scaffolding proteins referred to as MAGUKs, with PSD-95 being the most abundant in the postsynaptic density (PSD; Elias and Nicoll, 2007; Won et al., 2017; Zhu et al., 2016). It is postulated that these proteins contain “slots” that capture AMPARs freely diffusing on the cell surface (Choquet, 2018; Ehrlich and Malinow, 2004; Groc and Choquet, 2020; Schnell et al., 2002). Previous studies have found that deleting the PBM of TARP γ-8, the most abundant TARP in the hippocampus, prevents the trafficking of AMPARs to the synapses as well as LTP (Sheng et al., 2018). The biochemical findings showing that TARPs bind to the PDZ domains of PSD-95 (Chen et al., 2000; Zeng et al., 2019; Zhu et al., 2016) suggest that synaptic PSD-95 is the target for TARPs. However, while PSD-95 has long been proposed as a slot protein for AMPAR/TARP complexes, this has only been tested indirectly.

To directly test such a model and to address whether the TARP/PSD-95 interaction is a minimal core component of synaptic transmission and LTP, we have carried out a reconstitution experiment. We take advantage of the highly specific interaction between the *Drosophila* inactivation no afterpotential D (INAD) PDZ3 and the transient receptor potential (TRP) channel 15 carboxy-terminal residues (TRP15; Ye et al., 2016), an interaction which does not exist in mice. We replaced the PSD-95 PDZ2 domain with the INAD PDZ3 and replaced the TARP PBM with TRP15. Replacing these domains rendered each of these proteins functionally dead. However, co-expression of these chimeric proteins restored basal synaptic transmission and LTP. These results establish a minimal core interaction needed for AMPAR synaptic trafficking and LTP.

## RESULTS

**Figure 1.**
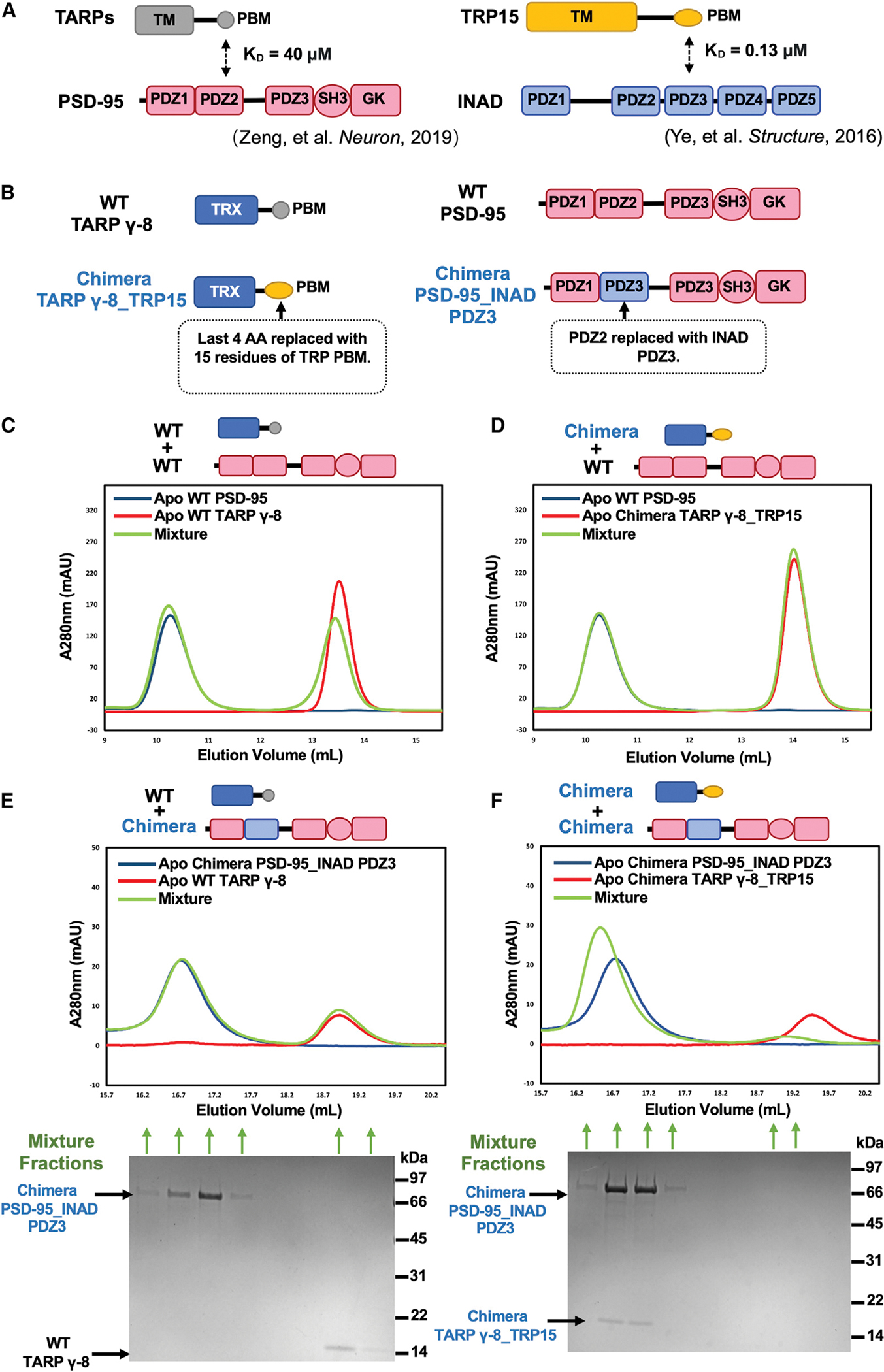
Protein designs for *in vitro* biochemical reconstitution (A) Schematic diagram showing two reported protein interacting pairs mediated by specific PDZ-PBM interaction. Left: TARP PBM weakly binds to PDZ2 of PSD-95 with K_D_ ~40 μM (Zeng et al., 2019). Right: *Drosophila* TRP PBM interacts with PDZ3 of INAD with K_D_ ~0.13 μM (Ye et al, 2016). (B) Detailed protein designs for *in vitro* biochemical reconstitution. Last 15 aa from mouse TARP γ-8 were fused to the C terminus of thioredoxin (TRX) to make “WT TARP γ-8.” A TARP γ-8 chimera was constructed by replacing the last 4 aa of WT TARP γ-8 with the last 20 aa of TRP to generate “chimera TARP γ-8_TRP15.” WT PSD-95 is composed of aa 61–724 of human PSD-95. A PSD-95 chimera was designed by replacing its PDZ2 with PDZ3 of INAD and termed as “PSD-95_INAD PDZ3.” (C) Analytical gel filtration chromatography showing that WT PSD-95 weakly interacts with WT TARP γ-8. 150 μM WT TARP γ-8 (colored in red), 50 μM WT PSD-95 (colored in blue), and their mixture (colored in green) were loaded onto a Superose 12 column (GE) with 100 μL injection volume. (D) Analytical gel filtration chromatography showing undetectable interaction between WT PSD-95 and chimera TARP γ-8_TRP15. 150 μM TARP γ-8_TRP15 (colored in red), 50 μM WT PSD-95 (colored in blue), and their mixture (colored in green) were loaded onto a Superose 12 column (GE) with 100 μL injection volume. (E) Analytical gel filtration chromatography (top panel) and SDS-PAGE with Coomassie blue staining analysis (bottom panel) showing no interaction between chimera PSD-95_INAD PDZ3 and WT TARP γ-8. 5 μM WT TARP γ-8 (colored in red), 5 μM PSD-95_INAD PDZ3 (colored in blue), and their mixture (colored in green) were loaded onto a Superose 6 column (GE) with 100 μL injection volume. For SDS-PAGE, 300 μL protein samples from each indicated fraction were first precipitated with acetone and then resolved with 20 μL 2X SDS loading buffer. Molecular weights are marked on the right side of the SDS-PAGE gel image. (F) Analytical gel filtration chromatography (top panel) and SDS-PAGE with Coomassie blue staining analysis (bottom panel) showing strong interaction between chimera PSD-95_INAD PDZ3 and chimera TARP γ-8_TRP15, indicated by co-elution of the two proteins in a single complex peak. 5 μM TARP γ-8_TRP15 (colored in red), 5 μM PSD-95_INAD PDZ3 (colored in blue), and their mixture (colored in green) were loaded onto a Superose 6 column (GE) with 100 μL injection volume. For SDS-PAGE, 300 μL protein samples from each indicated fraction were first precipitated with acetone and then resolved with 20 μL 2× SDS loading buffer. Molecular weights are marked on the right side of the SDS-PAGE gel image. Experiments shown in (C)–(F) have been performed at least three times, and the results are repeatable.

For our studies, we took advantage of the highly specific PDZ/target interaction between *Drosophila* INAD PDZ3 and the TRP channel tail (Ye et al., 2016). The strategy that we used is shown in Figure 1. The PBM of TARP γ-8 binds to the PDZ2 domain of PSD-95 with a K_D_ of 40 μM (Figure 1A, left panel) (Zeng et al., 2019). To isolate the contribution of the PDZ interaction to the binding of TARP γ-8 and PSD-95, we added the TARP PBM to a thioredoxin (TRX) domain (Figure 1B, top left panel). This TRX domain itself has no affinity for proteins in our study. Using analytical gel filtration chromatography, we demonstrated that this chimeric TRX-TARP PBM protein binds wild-type (WT) PSD-95 with a modest affinity (Figure 1C). We replaced the PBM of TRX-TARP PBM with that of the TRP channel (TRP15), creating the chimera TRX-TRP15 PBM (Figure 1B, bottom left panel). There was no detectable interaction between WT PSD-95 and TRX-TRP15 PBM (Figure 1D). The PBM of the TRP channel binds to the PDZ3 domain of INAD with a K_D_ of 0.13 μM (Figure 1A, right panel) (Ye et al., 2016). We next replaced the PDZ2 of PSD-95 with the PDZ3 of INAD, creating the chimera PSD-95_INAD PDZ3 (Figure 1B, bottom right panel). There was no detectable interaction between PSD-95_INAD PDZ3 and TRX-TARP PBM (Figure 1E). In contrast, a strong interaction is observed between chimera “PSD-95_INAD PDZ3” and chimera “TRX-TRP15 PBM,” as mixing PSD-95_INAD PDZ3 and TRX-TRP15 PBM results in a stable protein complex peak (Figure 1F).

**Figure 2.**
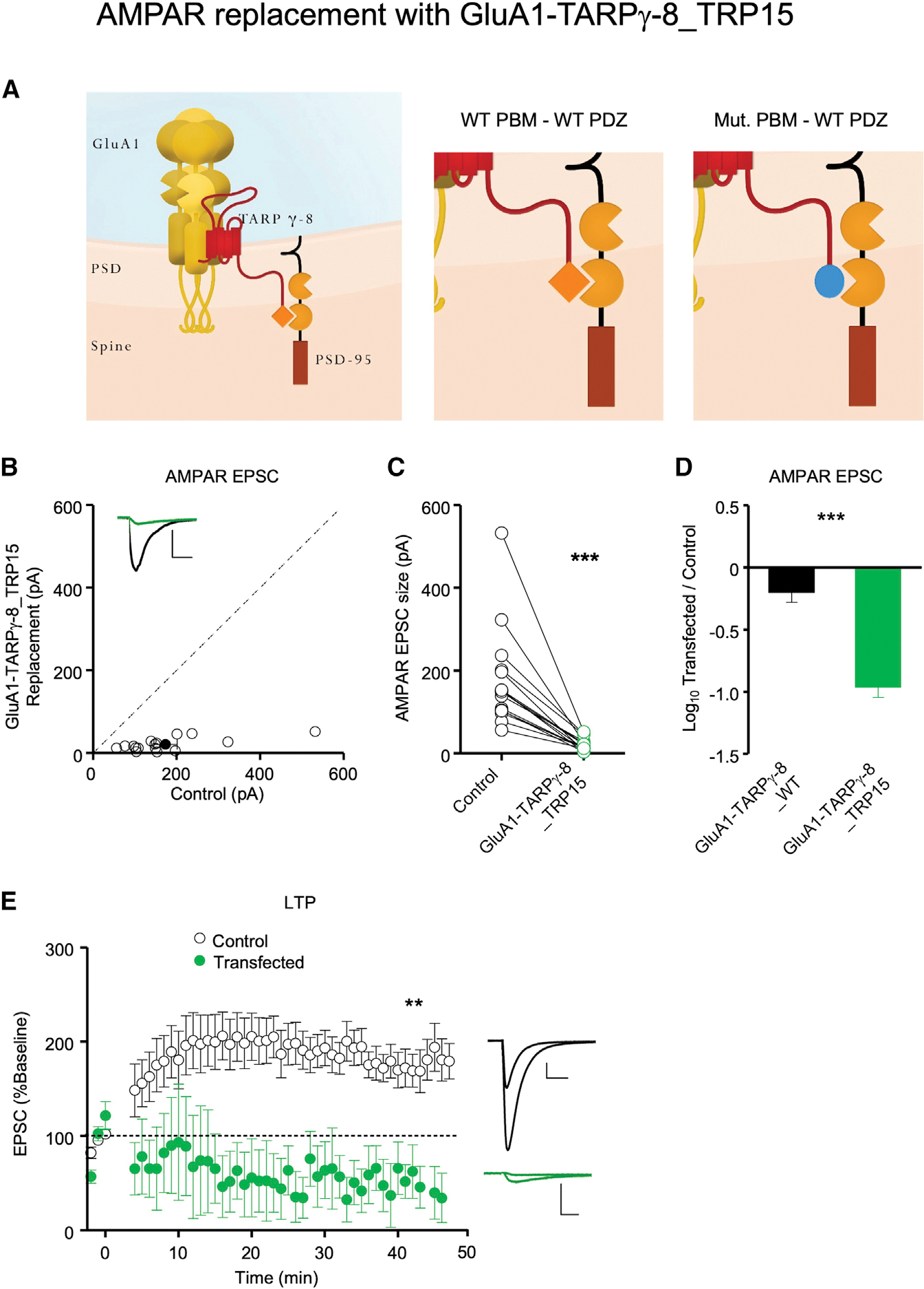
GluA1-TARP γ-8, but not GluA1-TARP γ-8_TRP15, rescues synaptic AMPAR transmission in AMPAR-null cells (A) (From left to right) Schematic of WT homomeric GluA1-TARP γ-8 AMPAR fusion protein in the PSD, schematic of the WT PDZ-PBM interaction, and schematic of the failed WT PDZ interaction with the mutant PBM in GluA1-TARP γ-8_TRP15 fusion protein replacement neurons. (B) GluA1-TARP γ-8_TRP15 replacement fails to rescue AMPAR EPSCs. Simultaneous dual whole-cell recordings were made from a transfected CA1 pyramidal neuron (green trace) and a neighboring WT one (black trace). Scatterplots showing amplitudes of AMPAR EPSCs for single pairs (open circles) and mean ± SEM (filled circle) of control and GluA1-TARP γ-8_TRP15 replacement neurons. Insets show representative EPSC traces (scale bars, 50 pA, 20 ms, n = 16 paired recordings). (C) Dot plots showing amplitudes of AMPAR EPSCs for single pairs of control (black) and GluA1-TARP γ-8_TRP15 (green) replacement neurons. Same data as in (B). (D) AMPAR replacement with GluA1-TARP γ-8 rescues AMPAR EPSCs (n = 19 paired recordings, reproduced from Zeng et al. [2019] for comparison), but replacement with GluA1-TARP γ-8_TRP15 (n = 16 paired recordings) does not. Bar graphs showing the mean log_10_ transfected/control EPSC ratio ± SEM. (E) Dual whole-cell paired LTP recordings were performed from a control CA1 neuron and a neighboring cell expressing Cre + GluA1-TARP γ-8_TRP15 in P16–P30 *Gria1–3*^fl/fl^ acute slices. Plots showing mean ± SEM. AMPAR EPSC amplitude of control (open circle) and Cre + GluA1-TARP γ-8_TRP15-expressing (green circle) CA1 pyramidal neurons. EPSCs for each experimental group are normalized to the mean AMPAR EPSC amplitude for that group, before LTP induction (minute 0). Insets show sample current traces before and at 40 min after LTP induction from control (black trace) and transfected (green trace) neurons. Scale bars: 50 pA, 20 ms. n = 9 control neuron recordings, n = 5 GluA1-TARP γ-8_TRP15 replacement neuron recordings. Data obtained from at least 3–4 mice per condition. Statistical significance was analyzed using the Wilcoxon signed-rank test in (C). Unpaired t test with Welch’s correction was used to compare groups in (D) and normalized EPSC values at 40 min in (E). **p < 0.01; ***p < 0.001.

Having successfully constructed chimeras that interact with one another specifically and with high affinity, we next turned to physiology. For our physiological assays, we adapted the AMPAR molecular replacement strategy we utilized in previous studies (Diaz-Alonso et al., 2017; Granger et al., 2013; Lu et al., 2009). All endogenous AMPARs were deleted by sparsely expressing the Cre recombinase in CA1 hippocampal pyramidal cells from *Gria1–3* triple-floxed mice (Lu et al., 2009). We next expressed the GluA1 AMPAR subunit tethered to TARP γ-8 (GluA1-TARP γ-8) on this AMPAR-null background (Sheng et al., 2018; Zeng et al., 2019). Previous studies have found that deleting the PBM of TARP γ-8 (GluA1-TARP γ-8D4) caused a failure to rescue AMPAR excitatory postsynaptic currents (EPSCs) (Sheng et al., 2018; Zeng et al., 2019), indicating an essential role of the TARP PBM in AMPAR trafficking. We first tested whether the GluA1-TARP γ-8_TRP15 chimera, where the PBM of TARP γ-8 was replaced with that of the *Drosophila* TRP channel tail (Figure 2A), could rescue AMPAR EPSCs. Using biolistic transfection, we co-expressed the Cre recombinase and the GluA1-TARP γ-8_TRP15 construct sparsely in hippocampal slice cultures from *Gria1–3* triple-floxed mice to achieve molecular replacement of endogenous AMPARs with GluA1 tethered to TARP γ-8_TRP15. In addition, we used acute slices from postnatal day 16 (P16)–P30 mice transfected by *in utero* electroporation. In both cases, this construct failed to rescue AMPAR responses (Figures 2B–2D), in contrast to the WT GluA1-TARP γ-8, reproduced for comparison from Zeng et al. (2019). There were no significant differences in results obtained using slice cultures and acute slices. Thus, we pooled the data from both acute slice and slice culture experiments in Figures 2B–2D. There was no change in the NMDAR EPSC in these experiments (Figure S1). We next examined LTP. Since, in our hands, LTP in slice culture is highly variable, we used the acute slice preparation described above. Here, GluA1-TARP γ-8_TRP15 replacement failed to rescue LTP (Figure 2E). The small responses remaining in these experiments are likely mediated by NMDARs (Diaz-Alonso et al., 2017; Sheng et al., 2018), which generally show a small and variable amount of LTP (reviewed in Nicoll [2017]) but, in this case, showed a slow run down. Given the inability of endogenous TARPs to access the TARP-tethered AMPAR (Sheng et al., 2018; Shi et al., 2009; Zeng et al., 2019), these experiments indicate that it is the lack of a TARP γ-8_TRP15 interaction with WT PSD-95 (Figure 1), which is naturally present in these experiments, that prevents the incorporation of AMPARs to the synapse and thus the rescue of AMPAR EPSCs.

**Figure 3.**
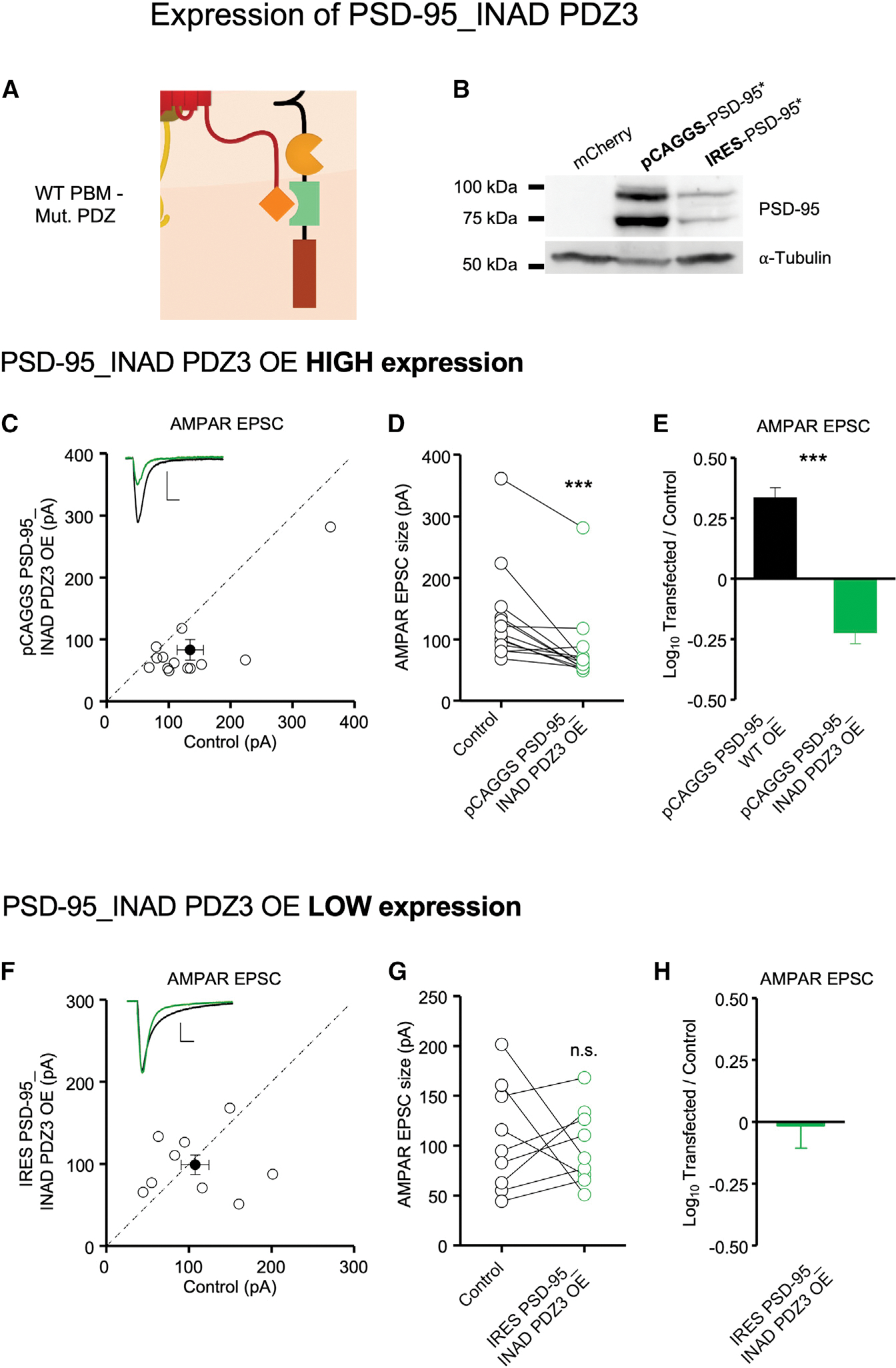
Effect of overexpressing PSD-95_INAD PDZ3 on AMPAR synaptic transmission (A) Schematic of the failed mutant PDZ interaction with the WT PBM in PSD-95_INAD PDZ3-expressing neurons. (B) PSD-95_INAD PDZ3 expression driven by a pCAGGS promoter is stronger than by an IRES promoter. Western blot images show 293T cell lysates stained for PSD-95 expression after 18 h of transfection. From left to right, cells were transfected with pCAGGS mCherry, pCAGGS-PSD-95_INAD PDZ3, and IRES PSD-95_INAD PDZ3. Alpha-tubulin is used as loading control. The absence of PSD-95 signal in the mCherry western blot validates antibody specificity. Each of the double bands may represent different PSD-95 cleavage products, such as before and after endoplasmic reticulum (ER) processing. n = 3 biological replicates. (C) Simultaneous dual whole-cell recordings were made from a transfected CA1 pyramidal neuron (green trace) and a neighboring WT one (black trace). Overexpression of pCAGGS PSD-95_INAD PDZ3 reduces AMPAR EPSC size. Scatterplots showing amplitudes of AMPAR EPSCs for single pairs (open circles) and mean ± SEM (filled circle) of control and pCAGGS PSD-95_INAD PDZ3 overexpression neurons. Insets show representative EPSC traces. n = 13 paired recordings (scale bars, 50 pA and 20 ms). (D) Dot plots showing amplitudes of AMPAR EPSCs for single pairs of control (black) and pCAGGS PSD-95_INAD PDZ3 (green) overexpression neurons. Same data as in (C). (E) Overexpression of wild-type PSD-95 potentiates synaptic AMPAR transmission 2.5-fold (n = 11 pairs, reproduced for comparison from Fukata et al. [2021]), but overexpression of pCAGGS PSD-95_INAD PDZ3 (n = 13 paired recordings) depresses synaptic AMPAR transmission. Bar graphs showing the mean log_10_ transfected/control EPSC ratio ± SEM. (F) Overexpression of IRES PSD-95_INAD PDZ3, which lowers the expression level relative to pCAGGS PSD-95_INAD PDZ3, does not affect EPSC size. Scatterplots showing amplitudes of AMPAR EPSCs for single pairs (open circles) and mean ± SEM (filled circle) of control (black trace) and IRES PSD-95_INAD PDZ3 (green trace) overexpression neurons. n = 9 paired recordings (scale bars, 50 pA and 20 ms). (G) Dot plots showing amplitudes of AMPAR EPSCs for single pairs of control (black) and IRES PSD-95_INAD PDZ3 (green) neurons. Same data as (F). (H) Overexpression of IRES PSD-95_INAD PDZ3 does not affect synaptic transmission. Bar graph showing the mean log_10_ transfected/control EPSC ratio ± SEM (n = 9 paired recordings). Data obtained from at least 3–4 mice per condition. Statistical significance was analyzed using the Wilcoxon signed-rank test in (D) and (G). Unpaired t test with Welch’s correction was used to compare groups in (E). ***p < 0.001; n.s., not significant.

Overexpressing PSD-95 causes a roughly 3-fold enhancement in AMPAR EPSCs with no change in the NMDAR EPSC (Beique and Andrade, 2003; Ehrlich and Malinow, 2004; Elias et al., 2008; Schnell et al., 2002; Stein et al., 2003; Figure 3E, black bar, reproduced for comparison from Fukata et al. [2021]). In marked contrast, expression of PSD-95_INAD PDZ3 in organotypic slice culture (Figure 3A) actually depressed synaptic responses (Figures 3C–3E), which we attribute to a dominant negative effect. In order to avoid this confounding effect in our reconstitution experiments, we decreased the expression level of PSD-95_INAD PDZ3 by inserting an internal ribosomal entry site (IRES) sequence upstream of the PSD-95 INAD PDZ3 cDNA (Figure 3B). In this case, in organotypic slice culture recordings, PSD-95_INAD PDZ3 had no effect on AMPAR responses (Figures 3F–3H). In these experiments, there was no change in the NMDAR EPSC (Figure S2).

**Figure 4.**
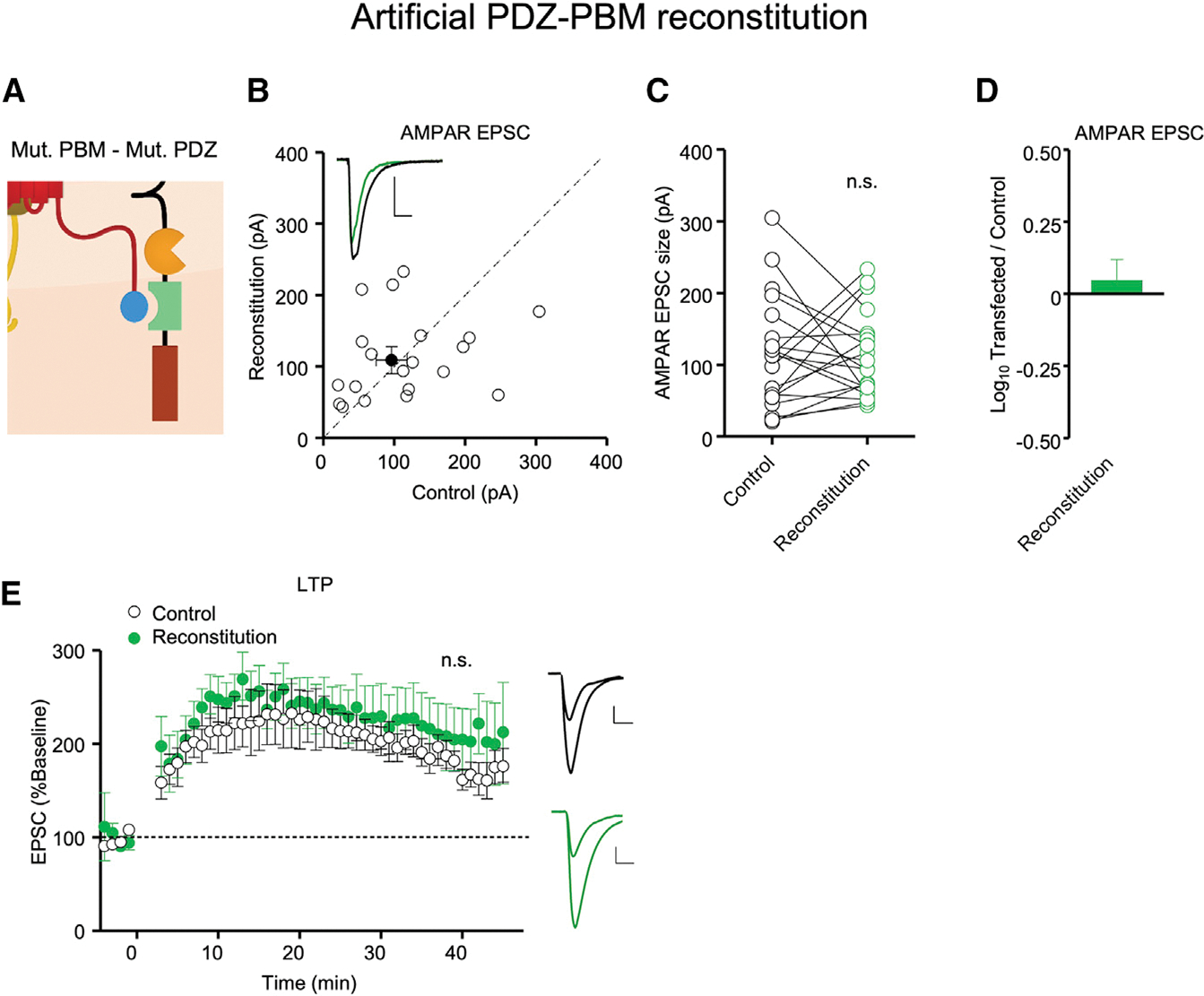
Co-expression of GluA1-TARP γ-8_TRP15 and PSD-95_INAD PDZ3 in AMPAR-null neurons reconstitutes synaptic AMPAR transmission and LTP Neurons in *Gria1–3*^fl/fl^ mice were co-transfected with Cre, GluA1-TARP γ-8_TRP15, and IRES PSD-95_INAD PDZ3. (A) Schematic of the artificial PDZ-PBM reconstitution in AMPAR-null neurons co-expressing GluA1-TARP γ-8_TRP15 and PSD-95_INAD PDZ3. (B) Simultaneous dual whole-cell recordings were made from a transfected CA1 pyramidal neuron (green trace) and a neighboring WT one (black trace). Scatterplots showing amplitudes of AMPAR EPSCs for single pairs (open circles) and mean ± SEM (filled circle) of control and Cre + GluA1-TARP γ-8_TRP15 + IRES PSD-95_INAD PDZ3 (reconstitution) transfected neurons. n = 20 recorded pairs (scale bars, 50 pA and 20 ms). (C) Dot plots showing amplitudes of AMPAR EPSCs for single pairs of control (black) and reconstitution (green) neurons. Same data as in (B). (D) Co-transfection of Cre, GluA1-TARP γ-8_TRP15, and IRES PSD-95_INAD PDZ3 in *Gria1–3*^fl/fl^ neurons rescues AMPAR EPSCs. Bar graph showing the mean log_10_ transfected/control EPSC ratio ± SEM. n = 20 recorded pairs. (E) Dual whole-cell paired LTP recordings were performed from a control CA1 neuron and a neighboring cell expressing Cre + GluA1-TARP γ-8_TRP15 + IRES PSD-95_INAD PDZ3 in P16–P30 *Gria1–3*^fl/fl^ acute slices. Plots showing mean ± SEM. AMPAR EPSC amplitude of control (open circle) and transfected (green circle) CA1 pyramidal neurons normalized to the mean AMPAR EPSC amplitude before LTP induction (minute 0). Insets show sample current traces before and 40 min after LTP induction from control (black trace) and transfected (green trace) neurons. n = 12 control neurons, n = 9 transfected neurons. Scale bars: 50 pA, 20 ms. Data obtained from at least 3–4 mice per condition. Statistical significance was analyzed using the Wilcoxon signed-rank test in (C). Normalized EPSC amplitudes at 40 min were compared using an unpaired t test with Welch’s correction in (E). n.s., not significant.

In a final series of experiments, we expressed GluA1-TARP γ-8_TRP15 together with PSD-95_INAD PDZ3 (Figure 4A), neither of which on their own were functional in mouse CA1 hippocampal pyramidal neurons (Figures 2 and 3), in AMPAR-null cells. As before, we recorded neurons in hippocampal slice culture as well as acute slices. We found no significant differences between the results in these two preparations, and the results were thus pooled. Basal AMPAR responses were fully restored (Figures 4B–4D). Furthermore, LTP was fully restored (Figure 4E). In these experiments, there was no change in the NMDAR EPSC (Figure S3). These results establish that the TARP γ-8/PSD-95 complex is a minimal core component of synaptic AMPAR trafficking and LTP.

## DISCUSSION

Research over the past decades has emphasized the central role of AMPAR trafficking in controlling basal excitatory synaptic transmission and LTP. PSD-95 has long been proposed as a synaptic slot protein necessary for anchoring AMPARs, but the underlying molecular mechanism remained mysterious. More recent research has focused on the TARP auxiliary subunits as intermediaries linking AMPARs to the synaptic MAGUK scaffolding proteins, such as PSD-95 (Chen et al., 2021; Greger et al., 2017; Hafner et al., 2015; Sheng et al., 2018; Straub and Tomita, 2012; Zeng et al., 2019). TARPs contain a C-terminal PBM, which binds to PDZ domains of PSD-95 (Chen et al., 2000; Dakoji et al., 2003; Schnell et al., 2002). Deleting the PBM disrupts both basal synaptic transmission and LTP (Sheng et al., 2018; Watson et al., 2021; Zeng et al., 2019). However, there are numerous synaptic proteins that contain PDZ domains, and it has yet to be definitively established whether the TARP PBM/PSD-95 PDZ interaction is, in fact, critical for the trafficking of AMPARs during LTP.

If the TARP/PSD-95 interaction is a minimal core component regulating AMPAR trafficking and LTP, we should be able to reconstitute basal synaptic transmission and LTP with an artificial TARP/PSD-95 complex. We took advantage of the highly specific interaction between the *Drosophila* INAD PDZ3 and the 15 carboxy-terminal residues in the TRP channel tail (TRP15) (Ye et al., 2016). The extreme C-terminal few residues of TRP15 correspond to the canonical PBM, and the rest of TRP15 forms a β-hairpin structure binding to a site on INAD PDZ3 that is away from its PBM binding groove. The requirement of TRP15 to form a high order β-hairpin structure, in addition to its canonical PBM, renders exquisite binding specificity between INAD PDZ3 and TRP15. We transplanted TRP15 into TARP γ-8, creating a TARP γ-8_TRP15 chimera. The exquisite specificity of the TARP γ-8_TRP15 chimera is highlighted by the fact that WT PSD95, which is naturally present at extremely high levels in the PSD (Chen et al., 2005; Cheng et al., 2006; Sheng and Hoogenraad, 2007), is insufficient to drive any synaptic incorporation of the GluA1-TARP γ-8_TRP15 tethered construct. We can therefore conclude that this chimera, when expressed on its own, was functionally dead. These findings reveal the necessity of a functional PBM-PDZ interaction for TARP-assisted AMPAR synaptic accumulation. We next transplanted the INAD PDZ3 into PSD-95, creating a PSD-95_INAD PDZ3 chimera. This chimera was also functionally dead when expressed on its own, demonstrating that the PBM-PDZ interaction is necessary for the substantial increase in AMPAR synaptic content upon PSD-95 overexpression. However, when we co-expressed these constructs, both basal synaptic transmission and LTP were restored. Thus, our results unequivocally establish a core protein-protein binding complex underlying AMPAR trafficking and LTP.

Previous work had suggested that TARPs and PSD-95 interact via a PDZ interaction (Schnell et al., 2002). However, there are major differences between the present study and previous work such that the experiments conducted in this paper provide fundamentally different insights. First, the mutation used in the previous study converts a type 1 PDZ interaction between TARP γ-2 and PSD-95 into a type 2 PDZ interaction. However, the distinction between these interactions is not robust (Ye and Zhang, 2013). Additionally, the interpretation of the previous results is limited because type 1/2 PDZ motifs and interactions are naturally occurring in the mouse proteome. Instead, the highly specific PDZ interaction between *Drosophila* INAD PDZ3 and the TRP channel tail used in the present study is a far superior system to attempt a reconstitution of AMPAR trafficking, as this is an interaction that does not occur in the mouse proteome. The sophistication of this system is demonstrated in the control experiment shown in Figure 3, where expressing the mutant PSD-95 by itself causes a decrease in synaptic transmission. This strong dominant negative effect on AMPAR-mediated synaptic transmission suggests that the PDZ2 motif of PSD-95 fully accounts for the interaction with TARPs. Second, in the Schnell et al. study (Schnell et al., 2002), all experiments involved overexpressing TARP constructs on a WT background. In the present study, we instead expressed GluA1-TARP constructs on an AMPAR-null background, thereby preventing the potential involvement of endogenous AMPAR complexes in the rescue. Third, since endogenous TARPs cannot access the TARP-tethered AMPAR (Sheng et al., 2018; Shi et al., 2009; Zeng et al., 2019), by tethering the AMPAR to the WT/mutant TARP γ-8, we are eliminating the potential involvement of other endogenous TARPs, in particular TARP γ-8, which is highly expressed in the hippocampus, as a variable. We thus feel that the present study greatly expands the Schnell et al. study (Schnell et al., 2002).

More generally, our results constitute strong evidence supporting an emerging model (Diaz-Alonso and Nicoll, 2021; Watson et al., 2021), establishing that the two main requisites for synaptic AMPAR complex trafficking are (1) intracellular interactions, dominated by the TARP PBM binding to PSD-95, and (2) the presence of intact extracellular AMPAR ATDs. Both are necessary conditions, which, combined, appear sufficient to enable synaptic accumulation of AMPARs constitutively and during LTP.

### Limitations of the study

While our results identify the TARP/PSD-95 PDZ as essential for AMPAR accumulation at the synapse, they do not exclude additional possible trafficking motifs. Our molecular replacement strategy, in which we replaced endogenous AMPARs with GluA1 tethered to TARP γ-8, was designed to focus specifically on TARP γ-8-based trafficking. However, previous studies reported only a partial loss of AMPAR EPSPs in the TARP γ-8 knockout (KO) mouse (Rouach et al., 2005). We find that acute single-cell deletion of TARP γ-8 with CRISPR on its own caused a 65% decrease in AMPAR EPSCs (Figure S4). It is unclear what mechanism controls the remainder of synaptic transmission. Previous studies have found thatdeleting TARP γ-2 or γ-3 individually or γ-2/γ-3 together had no effect on AMPAR EPSCs (Menuz et al., 2008). In addition, the deletion of TARP γ-3/γ-4 together has no effect on AMPAR EPSCs (Menuz et al., 2009). These findings raise the possibility that a component of synaptic transmission (~35%) may be TARP independent. Such a suggestion is in accord with previous observations (Sumioka et al., 2011; Watson et al., 2021). The nature of the interplay between TARP-dependent and TARP-independent AMPAR trafficking and LTP remains to be determined.

Our interpretation about the selectivity of the artificial PDZ-PBM reconstruction can potentially be affected by the different expression levels between WT PSD-95 (naturally present in the GluA1-TARP γ-8_TRP15 expression experiments; Figures 2B–2E) and mutant PSD-95 in the GluA1-TARP γ-8_TRP15/PSD-95_INAD PDZ3 reconstitution experiment (Figures 4B–4E). However, given the high endogenous levels of PSD-95 expression (Chen et al., 2005; Cheng et al., 2006; Sheng and Hoogenraad, 2007) and the expression of PSD-95_INAD PDZ3 under an IRES sequence, which substantially lowers its expression (Figure 3B), we estimate that the expression levels of PSD-95_INAD PDZ3 are similar to endogenous WT PSD-95 expression levels. We thus conclude that the absence of PDZ-PBM interaction, and not the abundance of WT PSD-95, is the main factor preventing the synaptic trafficking of GluA1-TARP γ-8_TRP15 in the absence of the complementary INAD PDZ3 mutation in PSD-95.

## STAR★METHODS

### RESOURCE AVAILABILITY

#### Lead contact

Further information, and requests for resources and reagents should be directed to and will be fulfilled by the lead contact, Roger Nicoll (roger.nicoll@ucsf.edu).

#### Materials availability

Plasmids generated in this study are available upon request. Requests for plasmids should be directed to and will be fulfilled by the lead contact, Roger Nicoll (roger.nicoll@ucsf.edu).

#### Data and code availability

- All data reported in this paper will be shared by the lead contact upon request.
- This paper does not report original code.
- Any additional information required to reanalyze the data reported in this paper is available from the lead contact upon request.

### EXPERIMENTAL MODEL AND SUBJECT DETAILS

#### Mice

*Gria1–3*^fl/fl^ C57BL6/N mice used in this study were genotyped as previously described (Lu et al., 2009). Slice cultures and acute slices were prepared from P6–P8 *Gria1–3*^fl/fl^ mouse pups or P16–30 mice, respectively, of either sex. For PSD-95_INAD PDZ3 overexpression experiments, slice cultures were prepared from P6–P8 wild-type CD-1 mouse pups of either sex. (Charles River Strain Code 022). All mice were maintained under a 12:12 h L/D schedule according to the University of California, San Francisco IACUC guidelines. All protocols were approved by the IACUC at University of California, San Francisco, in full compliance with NIH guidelines for humane treatment of animals.

### METHOD DETAILS

#### Recombinant proteins

Sequences encoding various proteins were generated using standard PCR-based methods, each cloned into a vector containing an N-terminal Trx-His_6_ or GB1-His_6_ tag followed by an HRV 3C cutting site. All constructs were confirmed by DNA sequencing. All recombinant proteins were expressed in Escherichia coli BL21-CodonPlus (DE3)-RIL (Agilent) in LB medium at 16°C overnight and protein expression was induced by 0.25 mM IPTG (final concentration) at OD_600_ between 0.6 and 0.8.

TRX-TARP PBM and TRX-TRP15 PBM were purified using a nickel-NTA agarose affinity column followed by Superdex 75 size-exclusion chromatography with a column buffer containing 50 mM Tris, pH 8.0, 100 mM NaCl, 1 mM EDTA, 2 mM DTT.

Trx-PSD-95 and GB1-PSD-95_INAD PDZ3 were purified using a nickel-NTA agarose affinity column followed by Superdex 200 size-exclusion chromatography with a column buffer containing 50 mM Tris, pH 8.0, 100 mM NaCl, 1 mM EDTA, 2 mM DTT. After cleavage by HRV 3C protease, a mono Q ion-exchange chromatography was added to remove the affinity tag and remaining contaminating proteins. Purified PSD-95 proteins were exchanged into a working buffer containing 50 mM Tris, pH 8.0, 100 mM NaCl, 1 mM EDTA, 2 mM DTT by a HiTrap desalting column.

#### Electrophysiology

For AMPAR replacement experiments, slice cultures were prepared from P6–P8 *Gria1–3*^fl/fl^ mouse pups as described previously (Stoppini et al., 1991) and biolistically transfected [Helios Gene Gun (Biorad)] at 1 DIV. Whole-cell voltage-clamp recordings were performed as described previously (Lu et al., 2009). Simultaneous dual recordings were taken from GFP-positive (transfected) neurons, as identified by nuclear (Cre-GFP) and cytoplasmic (pCAGGS-GluA1-TARP γ-8_TRP15-IRES-GFP) epifluorescence – and neighboring control CA1 pyramidal neurons at 14–22 DIV in organotypic slice cultures. For PSD-95_INAD PDZ3 overexpression and TARP γ-8 CRISPR deletion experiments, slice cultures were prepared from P6–P8 wild-type CD-1 mouse pups (Charles River Strain Code 022) as described previously (Stoppini et al., 1991) and biolistically transfected (Helios Gene Gun (Biorad)) at 1 DIV. For pCAGGS PSD-95 INAD PDZ3 overexpression experiments, neurons were co-transfected with pCAGGS PSD-95_INAD PDZ3 plasmid and pCAGGS mCherry plasmid. Neurons were identified using mCherry (red) signal. For TARP γ-8 deletion experiments, neurons were co-transfected with PX458 TARP γ-8 CRISPR/Cas9 plasmid and pCAGGS mCherry plasmid. Neurons were identified using mCherry (red) signal. Whole-cell voltage-clamp recordings were performed as described previously (Lu et al., 2009).

For recording, slices were placed in a perfusion chamber on an Olympus BX51WI upright microscope and perfused at 2.5 mL/min with artificial cerebrospinal fluid (aCSF) containing (in mM): 119 NaCl, 2.5 KCl, 1 NaH_2_PO_4_, 26.2 NaHCO_3_ and 11 glucose, 4 CaCl_2_, 4 MgSO_4_, 0.1 picrotoxin, 0.02 bicuculline and 2 μM 2-chloroadenosine. The aCSF was bubbled with 95% O_2_ and 5% CO_2_, and osmolarity was adjusted to 302–305 mOsm. The internal whole-cell recording solution contained (in mM) 135 CsMeSO_4_, 8 NaCl, 10 HEPES, 0.3 EGTA, 5 QX-314, 4 Mg-ATP, and 0.3 Na-GTP and 0.1 spermine. Osmolarity was adjusted to 290–292 mOsm, and pH at 7.3–7.4. Synaptic responses were evoked by stimulating with a bipolar stimulation electrode (Microprobes) placed in the *stratum radiatum*, and responses were evoked at 0.2 Hz. For LTP recordings, responses were evoked at 0.1 Hz.

To ensure stable recording, membrane holding current, input resistance, and pipette series resistance were monitored throughout the experiment. Data were gathered through a MultiClamp 700B amplifier (Axon Instruments), filtered at 2 kHz, and digitized at 10 kHz. AMPAR-mediated responses were isolated by voltage-clamping the cell at −70 mV, whereas NMDARs were recorded at +40 mV, with amplitudes taken 150 ms after stimulation to avoid contamination by AMPAR current.

#### *In vivo* AMPAR replacement

For AMPAR replacement experiments *in vivo*, *Gria1–3*^fl/fl^ mouse brains were transfected in utero at E15.5. For Figure 2, mouse brains were transfected with pFUGW-Cre:GFP and pCAGGS-GluA1-TARP γ-8_TRP15 constructs. For Figure 4, neurons were co-transfected with pFUGW-Cre:GFP, pCAGGS-GluA1-TARP γ-8_TRP15 and pCAGGS-IRES-PSD-95_INAD PDZ3.

In utero electroporation was performed as follows: E15.5 pregnant *Gria1–3*^fl/fl^ mice were anesthetized with 2% isoflurane in O_2_. For analgesia, 0.05 mg/kg buprenorphine (Reckitt Benckiser Healthcare) and 2 mg/kg meloxicam (Boehringer Ingelheim) were injected subcutaneously after induction of anesthesia. Embryos were then exposed out of the abdominal cavity and 1.5 μL of mixed plasmid DNA were injected into the lateral ventricle using a beveled glass micropipette. For Figure 2, pFUGW-Cre:GFP (0.5 μg/μL final concentration) was mixed with pCAGGS-GluA1-TARP γ-8_TRP15-IRES GFP (1.5 μg/μL final concentration). For Figure 4, pFUGW-Cre:GFP (0.5 μg/μL final concentration) was mixed with pCAGGS-GluA1-TARP γ-8_TRP15-IRES GFP (1.5 μg/μL final concentration) and pCAGGS-IRES-PSD-95_INAD PDZ3 (1.5 μg/μL final concentration). 0.1% Fast Green (Sigma Aldrich) was added to the DNA mix to help visualization of the injection site. After injection, embryos were electroporated with 5 pulses of 40 V during 50 msec, delivered at 1 Hz, using platinum tweezertrodes (BTX Harvard Apparatus) with a square-wave pulse generator (BTX Harvard Apparatus). To maximize electroporation of the hippocampus, the positive electrode was placed in the lower right hemisphere and the negative electrode placed in the upper left hemisphere. After electroporation, the embryos were placed into the abdominal cavity and the abdominal muscle and skin were sutured. Pregnant females were maintained on a heated pad and monitored during the surgical procedure and the post-surgery period.

#### Acute slice electrophysiology and LTP induction

300 μm transverse acute slices were cut from P16–30 electroporated mice using a Microslicer DTK-Zero1 (Ted Pella) in chilled high sucrose cutting solution containing (in mM): 2.5 KCl, 7 MgSO_4_, 1.25 NaH_2_PO_4_, 25 NaHCO_3_, 7 glucose, 210 sucrose, 1.3 ascorbic acid. The slices were then incubated for 30 min at 34°C in aCSF containing (in mM): 119 NaCl, 2.5 KCl, 1 NaH_2_PO_4_, 26.2 NaHCO_3_, 11 glucose, 2.5 CaCl_2_ and 1.3 MgSO_4_. The slices were then transferred to the recording chamber, and 0.1 mM picrotoxin and 0.02 mM bicuculline were added to the aCSF for recordings. Simultaneous dual recordings were taken from GFP positive (transfected) neurons, as identified by nuclear (Cre-GFP) and cytoplasmic (GluA1-TARP γ-8_TRP15-IRES-GFP) epifluorescence, and neighboring control CA1 pyramidal neurons. LTP was induced by stimulating Schaffer collateral axons at 2 Hz for 90 s while clamping the cell at 0 mV, after recording a ~3 min baseline, but not more than 5 min after breaking into the cell. Synaptic responses before (baseline) and after LTP induction were evoked at 0.1 Hz. In some cases, one of the two cells was lost at some point during the LTP experiment. Recordings were considered until that point, which result in larger SEM in later stages of the LTP experiment. In cases where only one cell was lost, the remaining cell was considered for the averages. Unpaired statistics were used as a result to determine statistical significance of the LTP experiment.

#### Immunoblot

18–48 h post-transfection with Lipofectamine 2000, 293 T cells were washed in PBS, pelleted and re-suspended directly in SDS-containing sample buffer. All samples were run in a PAGE-SDS electrophoresis. PVDF membranes were blocked with 5% blotting grade nonfat milk in tris-buffered saline buffer with 0.1% tween 20. PVDF membranes were then incubated with primary and secondary antibodies, and then imaged.

### QUANTIFICATION AND STATISTICAL ANALYSIS

Statistical analyses were performed using the two-tailed unpaired t test with Welch’s correction for all experiments involving unpaired data and the two-tailed Wilcoxon signed-rank test for all experiments using paired whole-cell data, including all synaptic replacement and synaptic overexpression data. LTP data were gathered from pairs of control and experimental neurons; however, some cells were lost during the experiment. Consequently, the resulting datasets are a mix of interleaved and paired data, and thus comparisons were made using the unpaired t test with Welch’s correction. Summarized data were presented in figures as mean ±SEM with n values representing the number of cells in each dataset. All statistical significance was set as *p < 0.05, **p < 0.01, and ***p < 0.001.

## Supplementary Material

1

## Figures and Tables

**KEY RESOURCES TABLE:** 

REAGENT or RESOURCE Antibodies	SOURCE	IDENTIFIER

Antibodies		

TARP γ-8 antibody, Rabbit	ThermoFisher Scientific	Cat# PA5-77354; RRID:AB_2735560
β-actin, Mouse	Sigma-Aldrich	Cat# A5441; RRID:AB_476744
LICOR IRDye 680 LT, Goat anti-Rabbit	Neta Scientific	Cat# LIC-926-68021; RRID:AB_10706309
PSD-95	Synaptic Systems	Cat# 124011; RRID:AB_10804286
Alpha-tubulin	Millipore Sigma	Cat# T9026; RRID:AB_477593
Horse anti-Mouse-HRP	Vector Laboratories	Cat# PI-2000-1; RRID:AB_2336177
LICOR IRDye 800 CW, Goat anti-Mouse	Neta Scientific	Cat# LIC-926-32210; RRID:AB_621842

Bacterial and virus strains		

Escherichia coli BL21-CodonPlus (DE3)-RIL Competent Cells	Agilent	Cat# 230245
Stellar Competent Cells	Takara Bio	Cat# 636763

Chemicals, peptides, and recombinant proteins		

PSD-95_INAD PDZ3	This paper	N/A
GluA1-TARP γ-8_TRP15	This paper	N/A
TRX-TARP WT PBM	This paper	N/A
TRX-TARP TRP PBM	This paper	N/A
PSD-95 Full length (aa 1M-724L, UniProt: P78352-1)	Zeng et al., (2019)	N/A
TARP γ-8	Rouach et al. (2005)	N/A
Blotting-Grade Nonfat Milk	Bio-Rad	Cat# 1706404
Tween-20	Acros Organics	Cat# HV-88248-24
Lipofectamine™ 2000 Transfection Reagent	ThermoFisher Scientific	Cat# 11668019

Critical commercial assays		

Helios Gene Gun Kit	Bio-Rad	Cat# 1652411
Helios Cartridge Kit	Bio-Rad	Cat# 1652440

Experimental models: Cell lines		

293T Cells	ATCC	CRL-3216

Experimental models: Organisms/strains		

C57BL6/N Gria1-3 fl/fl mice	Lu et al. (2009)	N/A

Oligonucleotides		

TARP γ-8 CRISPR: GATGACGGACCACCCCATCG	This paper	N/A

Recombinant DNA		

Plasmid for recombinant protein expression: PSD-95_INAD PDZ3 HIGH expression	This paper	N/A
Plasmid for recombinant protein expression: PSD-95_INAD PDZ3 LOW expression	This paper	N/A
Plasmid for TARP γ-8 CRISPR expression: pSpCas9(BB)-2A-GFP (PX458)	Genome engineering using the CRISPR-Cas9 system. Ran et al. (2013)	Addgene_48138
Plasmid for recombinant protein expression: GluA1-TARP γ-8_TRP15	This paper	N/A
Software and algorithms		
PyMOL	PyMOL	http://pymol.sourceforge.net/
ImageJ	NIH	https://imagej.nih.gov/ij/
Prism	GraphPad	https://www.graphpad.com/scientific-software/prism/
Igor Pro	Wavemetrics	https://www.wavemetrics.com/products/igorpro
Benchling CRISPR guideRNA design tool	Benchling	https://www.benchling.com/
Origin7.0	OriginLab	https://www.originlab.com/

## Highlights

- Replacing the TARP PDZ binding motif with an exogenous version kills TARP function
- PSD-95 with an exogenous PDZ2 domain is functionally dead when expressed alone
- Complementary PSD-95 and TARP chimeras restore synaptic transmission and LTP
- The TARP/PSD-95 interaction is necessary for synaptic AMPAR trafficking and LTP
