# Supplementary material for: Long-term potentiation reconstituted with an artificial TARP/PSD-95 complex
Source: Cell Rep. Author manuscript; Available in PMC 2022 Dec 28. (PMC9797105; doi:10.1016/j.celrep.2022.111483)
Supplement: 1 [file NIHMS1842063-supplement-1.pdf]

Cell Reports, Volume *41*

Supplemental information

Long-term potentiation reconstituted

with an artificial TARP/PSD-95 complex

Anagh Sinha Ravi, Menglong Zeng, Xudong Chen, Gerardo Sandoval, Javier Diaz-Alonso, Mingjie Zhang, and Roger A. Nicoll

AMPAR replacement with GluA1-TARPγ-8_TRP15

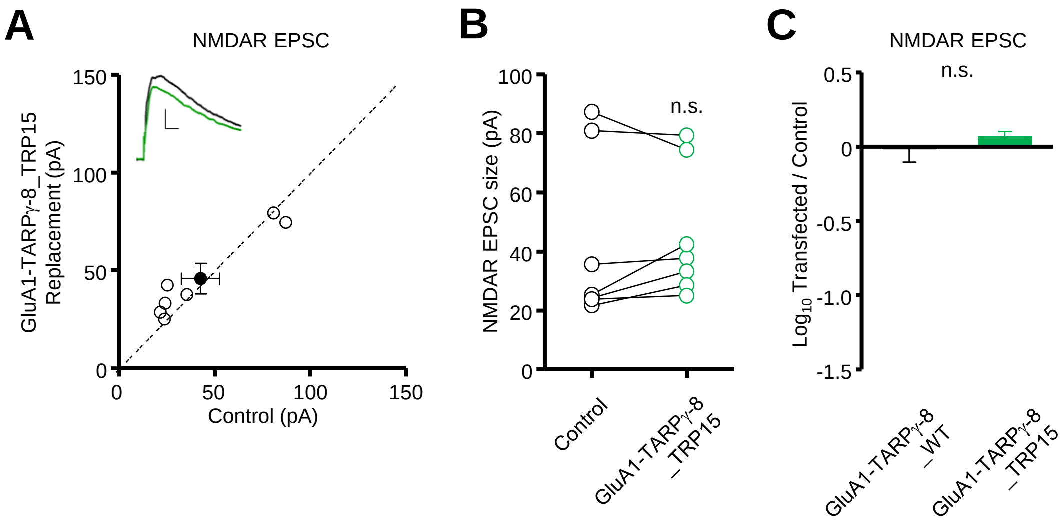

Figure S1: Expression of GluA1-TARP γ-8_TRP15 does not affect NMDAR EPSCs in AMPAR-null cells. Related to Figure 2. (A) Simultaneous dual whole-cell recordings were made from a transfected CA1 pyramidal neuron (green trace) and from a neighboring wild-type one (black trace). GluA1-TARP γ-8_TRP15 replacement has no effect on NMDAR EPSCs. Scatterplots showing amplitudes of NMDAR EPSCs for single pairs (open circles) and mean ± SEM (filled circle) of control and GluA1-TARP γ-8_TRP15 replacement neurons. Insets show representative EPSC traces (scale bars, 50 pA, 20 ms, n = 7 paired recordings). (B) Dot-plots showing amplitudes of NMDAR EPSCs for single pairs of control (black) and GluA1-TARP γ-8_TRP15 (green) replacement neurons. (C) AMPAR replacement with either GluA1-TARP γ-8 or GluA1-TARP γ-8_TRP15 does not affect NMDAR EPSCs (n = 7 paired recordings, reproduced from (Zeng et al., 2019) for comparison). Bar graphs showing the $\log_{10}$ transfected/control EPSC ratio ± SEM. Statistical significance was analyzed using the Wilcoxon signed-rank test in (B). Unpaired t-test with Welch's correction was used to compare relevant groups in (C). ns, not significant.

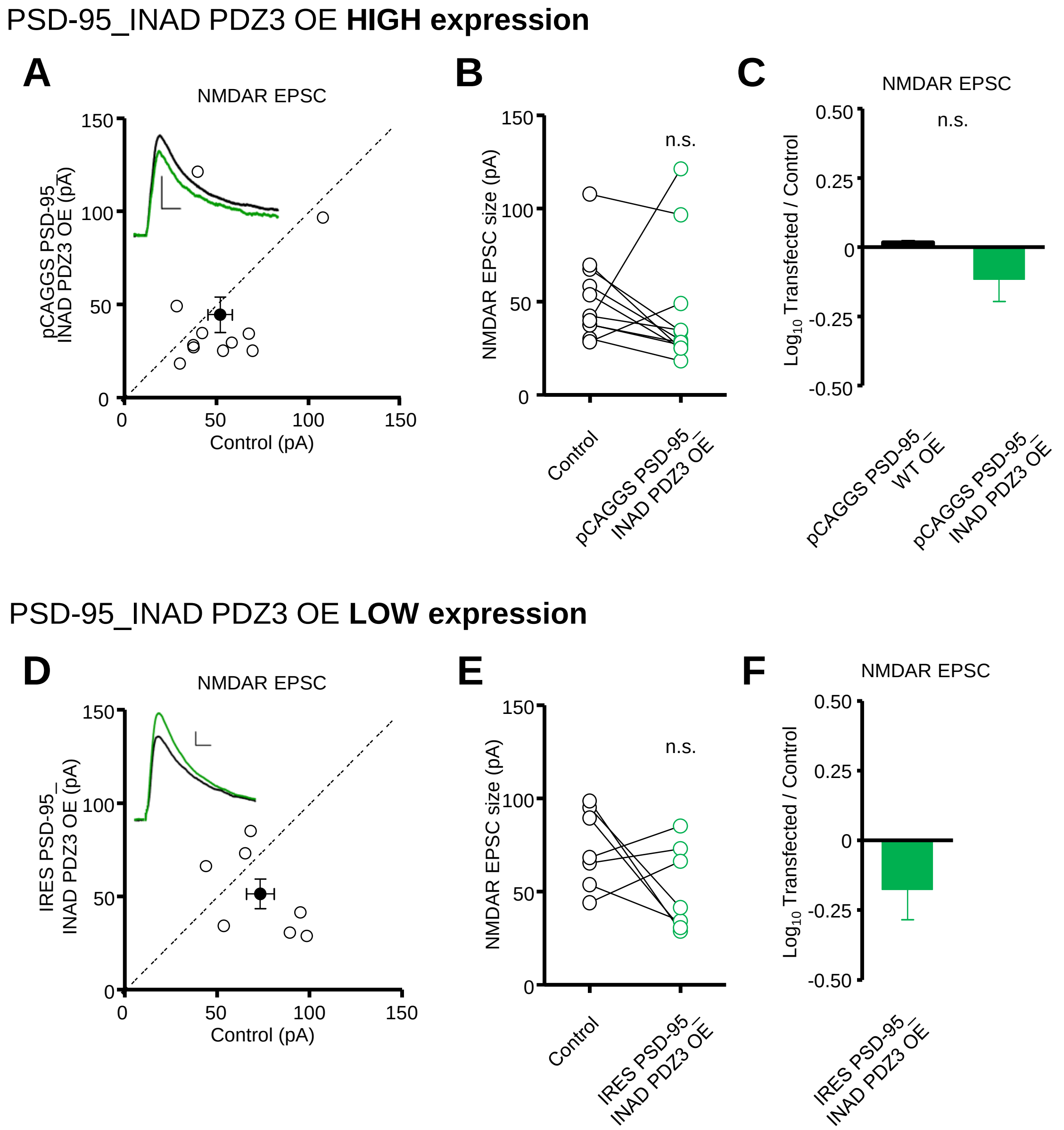

Figure S2: Overexpressing PSD-95_INAD PDZ3 does not affect NMDAR synaptic transmission. Related to Figure 3. (A) Overexpression of pCAGGS PSD-95_INAD PDZ3 does not affect NMDAR EPSC size. Scatterplots showing amplitudes of NMDAR EPSCs for single pairs (open circles) and mean ± SEM (filled circle) of control (black trace) and pCAGGS PSD-95_INAD PDZ3 overexpression (green trace) neurons. Insets show representative EPSC traces (scale bars, 50 pA and 20 ms). n = 8 paired recordings. (B) Dot-plots showing amplitudes of NMDAR EPSCs for single pairs of control (black) and pCAGGS PSD-95_INAD PDZ3 (green) overexpression neurons. (C) Overexpressing WT PSD-95 (n = 8 paired recordings, reproduced from (Fukata et al., 2021) for comparison) or pCAGGS PSD-95_INAD PDZ3 OE does not affect NMDAR EPSCs. Bar graphs showing the log_{10} transfected/control EPSC ratio ± SEM. (D) Overexpression of IRES PSD-95_INAD PDZ3 does not affect NMDAR EPSC size. Scatterplots showing amplitudes of NMDAR EPSCs for single pairs (open circles) and mean ± SEM (filled circle) of control (black trace) and IRES PSD-95_INAD PDZ3 overexpression (green trace) neurons. Insets show representative EPSC traces (scale bars, 50 pA and 20 ms). n = 7 paired recordings. (E) Dot-plots showing amplitudes of NMDAR EPSCs for single pairs of control (black) and IRES PSD-95_INAD PDZ3 (green) neurons. (F) Overexpression of IRES PSD-95_INAD PDZ3 does not affect NMDAR EPSCs. Bar graphs showing the log_{10} transfected/control EPSC ratio ± SEM. Statistical significance was analyzed using the Wilcoxon signed-rank test in (B, E). Unpaired t-test with Welch's correction was used to compare relevant groups in (C). ns, not significant.

Artificial PDZ-PBM reconstitution

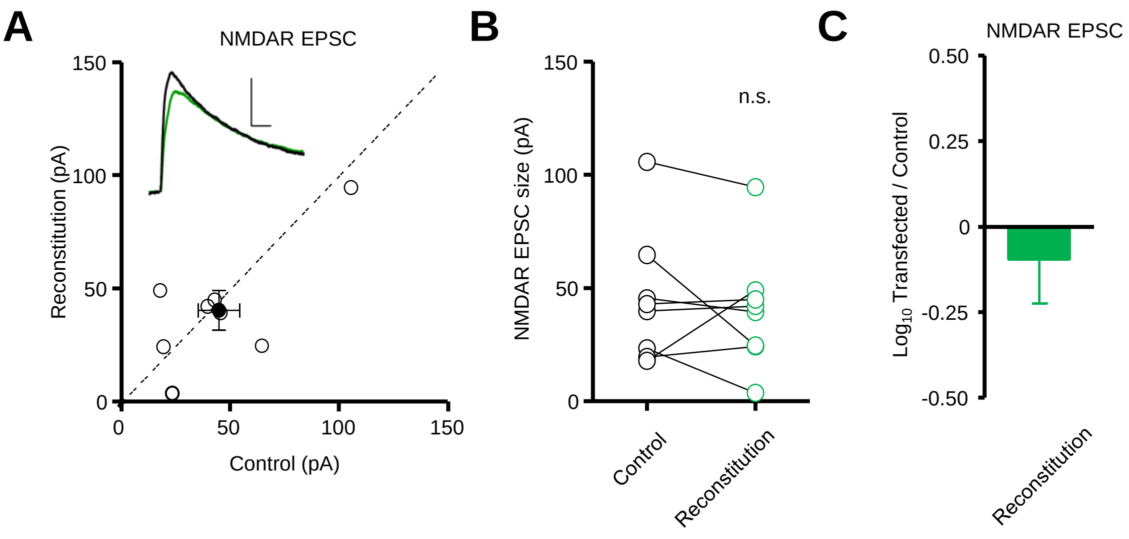

Figure S3: Co-expression of GluA1-TARP γ-8_TRP15 and PSD-95_INAD PDZ3 in AMPAR-null neurons does not affect NMDAR transmission. Related to Figure 4. (A) Simultaneous dual whole-cell recordings were made from a transfected CA1 pyramidal neuron (green trace) and a neighboring wild-type one (black trace). Insets show representative EPSC traces (scale bars: 50 pA, 20 ms). Scatterplots showing amplitudes of NMDAR EPSCs for single pairs (open circles) and mean ± SEM (filled circle) of control and Cre + GluA1-TARP γ-8_TRP15 + IRES PSD-95_INAD PDZ3 (Reconstitution) transfected neurons. n = 8 recorded pairs. (B) Dot-plots showing amplitudes of NMDAR EPSCs for single pairs of control (black) and Reconstitution (green) neurons. (C) Co-transfection of Cre, GluA1-TARP γ-8_TRP15, and IRES PSD-95_INAD PDZ3 in *Gria1-3*$^{fl/fl}$ neurons does not affect NMDAR EPSCs. Bar graphs showing the $\log_{10}$ transfected/control EPSC ratio ± SEM. Statistical significance was analyzed using the Wilcoxon signed-rank test in (B). n.s., not significant.

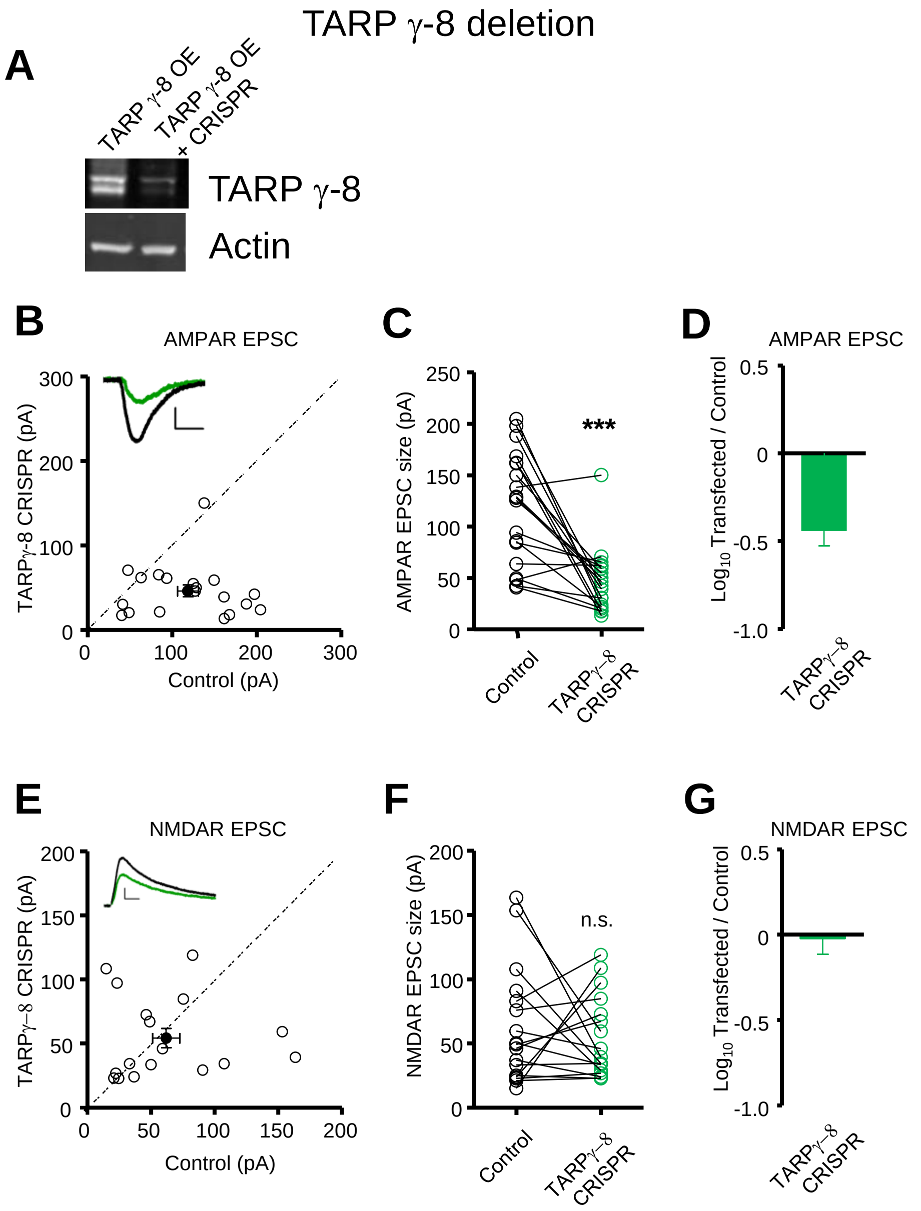

Figure S4: Acute CRISPR deletion of TARP γ-8 in slice culture reduces AMPAR EPSCs but does not affect NMDAR EPSCs. Related to Figure 2 and 4. (A) TARP γ-8 CRISPR deletion is effective in 293T cells. 293T cells were simultaneously transfected for 48 hours with TARP γ-8 overexpression (OE) plasmid and Cas9 alone (left) or TARP γ-8 OE plasmid with TARP γ-8 CRISPR and Cas9 (right). Western blot comparison of protein levels demonstrates effective deletion of TARP γ-8, with β-Actin used as a loading control. This experiment contained 2 technical replicates. The experiment was repeated once, and the results were repeatable. (B) Simultaneous dual whole-cell recordings were made from a transfected CA1 pyramidal neuron (green trace) and a neighboring wild-type one (black trace). Scale bars: 50 pA, 20 ms. Scatterplots showing amplitudes of AMPAR EPSCs for single pairs (open circles) and mean ± SEM (filled circle) of control and TARP γ-8 CRISPR + Cas9 transfected neurons. n = 19 recorded pairs. (C) Dot-plots showing amplitudes of AMPAR EPSCs for single pairs of control (black) and TARP γ-8 CRISPR + Cas9 (green) neurons. (D) Bar graphs showing the $\log_{10}$ transfected/control AMPA EPSC ratio ± SEM. (E) Simultaneous dual whole-cell recordings were made from a transfected CA1 pyramidal neuron (green trace) and a neighboring wild-type one (black trace). Scale bars: 50 pA, 20 ms. Scatterplots showing amplitudes of NMDAR EPSCs for single pairs (open circles) and mean ± SEM (filled circle) of control and TARP γ-8 CRISPR + Cas9 transfected neurons. n = 17 recorded pairs. These are from the same recordings made in A, minus two cells which were lost before the NMDAR response could be recorded. (F) Dot-plots showing amplitudes of NMDAR EPSCs for single pairs of control (black) and TARP γ-8 CRISPR + Cas9 (green) neurons. (G) Bar graphs showing the $\log_{10}$ transfected/control NMDA EPSC ratio ± SEM. Statistical significance was analyzed using the Wilcoxon signed-rank test in (C, F). *** $P < 0.001$; n.s. not significant.
